# Prostaglandin E_2_-EP3 Axis in Fine-Tuning Excessive Skin Inflammation by Restricting Dendritic Cell Functions

**DOI:** 10.1371/journal.pone.0069599

**Published:** 2013-07-29

**Authors:** Noriko Shiraishi, Takashi Nomura, Hideaki Tanizaki, Saeko Nakajima, Shuh Narumiya, Yoshiki Miyachi, Yoshiki Tokura, Kenji Kabashima

**Affiliations:** 1 Department of Dermatology, University of Environmental and Occupational Health, Kitakyushu, Japan; 2 Department of Dermatology, Kyoto University Graduate School of Medicine, Kyoto, Japan; 3 Department of Pharmacology, Kyoto University Graduate School of Medicine, Kyoto, Japan; 4 Department of Dermatology, Hamamatsu University School of Medicine, Hamamatsu, Japan; Academic Medical Center, Netherlands

## Abstract

Prostaglandin E_2_ (PGE_2_) is produced in the skin and is suggested to play a role in the regulation of cutaneous immune homeostasis and responses. However, the multifaceted functions of PGE_2_ continue to elude our understanding, especially because of the multiplicity of PGE_2_ receptors—EP1, EP2, EP3, and EP4. While cAMP-elevating EP4 is known to activate the functions of cutaneous dendritic cells (DCs), including Langerhans cells (LCs) and dermal DCs, the role of cAMP-suppressing EP3 in this process remains unknown. Here we demonstrated that an EP3 receptor selective agonist, ONO-AE-248, inhibited chemotaxis and co-stimulatory molecule expressions of DCs *in vitro*. A suboptimal dose of antigen was sufficient to induce contact hypersensitivity in EP3-deficient mice. Intriguingly, EP3 deficiency did not impair skin inflammation at all when the antigen dose was sufficiently high. EP3 limited the functions of cutaneous DCs only when the antigen dose was low. In contrast to EP4, the observed unappreciated function of EP3 may stabilize the cutaneous DCs to halt the impetuous response to a suboptimal dose of antigen. Taken together, PGE_2_-EP3 signaling is essential for fine-tuning excessive skin inflammation by restricting DC functions.

## Introduction

The skin is continually exposed to multiple foreign antigens throughout life and it exerts antigen-specific immune responses in which skin dendritic cells (DCs) play an important role as antigen-presenting cells (APCs) [1]. DCs in the skin are roughly divided into three subsets: Langerhans cells (LCs) in the epidermis and Langerin^+^ or Langerin^-^ DCs in the dermis [2]. These cutaneous DCs play a central role in the immunity and tolerance of the skin. Responding to foreign antigen, cutaneous DCs migrate to draining lymph nodes undergoing terminal differentiation and maturation. Matured cutaneous DCs then activate naive T cells to induce antigen-specific effector/memory T cells in the lymph nodes [3]. The migration and maturation of cutaneous DCs are, therefore, crucial for the initiation of specific immune responses in the skin.

Lines of evidence suggest that prostanoids, including prostaglandins (PGs), engage in this DC alteration step [4,5]. On exposure to physiological or pathological stimuli, arachidonic acid is liberated from cell membrane phospholipids and is converted to prostanoids, including PGD_2_, PGE_2_, PGF_2α_, PGI_2_, and thromboxane A_2_, through cyclooxygenases-mediated oxygenation followed by respective synthases. Prostanoids are produced in large amounts during inflammation and they exert complicated actions, including swelling, pain sensation, and fever generation. Among the prostanoids, PGD_2_ and PGE_2_ are abundantly produced in the skin during the elicitation phase of contact hypersensitivity (CHS)—a murine model for allergic contact dermatitis [3,6,7]. Therefore, it is of interest to evaluate the roles of PGD_2_ and PGE_2_ on DC functions.

It has been reported that PGD_2_ suppresses cutaneous DC functions via DP1 receptor [8], while it enhances these functions via CRTH2 [9]. PGE_2_ is produced abundantly in the skin on exposure to antigen [10], and is supposed to play a key role in determining the direction of immune response. Indeed, PGE_2_ affects an immune response differently in a context-dependent fashion, showing some inconsistency at first glance. This contradictory effect is partially explained by the complexity of the four subtypes for the EP—the type E prostanoid receptors for PGE_2_, i.e., EP1, EP2, EP3, and EP4, each of which couples a different type of G protein. EP1 mediates the elevation of intracellular Ca^2+^ concentration to promote Th1 differentiation [11]. On the other hand, EP2 and EP4 couple Gs protein that activates the cyclic adenosine monophosphate (cAMP)-dependent pathway by activating adenylate cyclase. EP2 is a potent suppressor of T cell proliferation *in vitro* [12,13]. EP4 suppresses T cell proliferation *in vitro* [12–14] and reinforces immunosuppression by expanding the number of Treg cells *in vivo* [15]. However, in a contradictory manner, EP4 also initiates the CHS response by inducing the migration and maturation of cutaneous DCs [10]. EP3 couples the Gi protein that inhibits cAMP-dependent pathways. We previously demonstrated that EP3 inhibited CHS by restraining keratinocytes from producing CXCL1, a neutrophil-attracting chemokine ligand CXCL1 [16]. EP3 is highly expressed in cutaneous DCs; however, the role of EP3 in APCs has not been studied in detail.

In this study, we demonstrated that EP3 downregulated the functions of DCs and that CHS was induced in m*Pger3* (EP3)-deficient (EP3KO) mice upon exposure to suboptimal doses of antigens. Our results suggest that EP3 signaling inhibits undesired skin inflammation by limiting the maturation and migration of cutaneous DCs.

## Results

### Expression of EP3 in bone marrow-derived DCs

EP subtypes are differentially expressed in the organs depending on the cell types. While the role of cAMP-elevating EP4 is known to enhance the functions of cutaneous DCs, the role of cAMP-decreasing EP3 remains unclear. It has been reported that EP3 is widely expressed in immune cells in mice [17], such as DCs [17], macrophages [18], and B cells [19], but not T cells [20].

**Figure 1:**
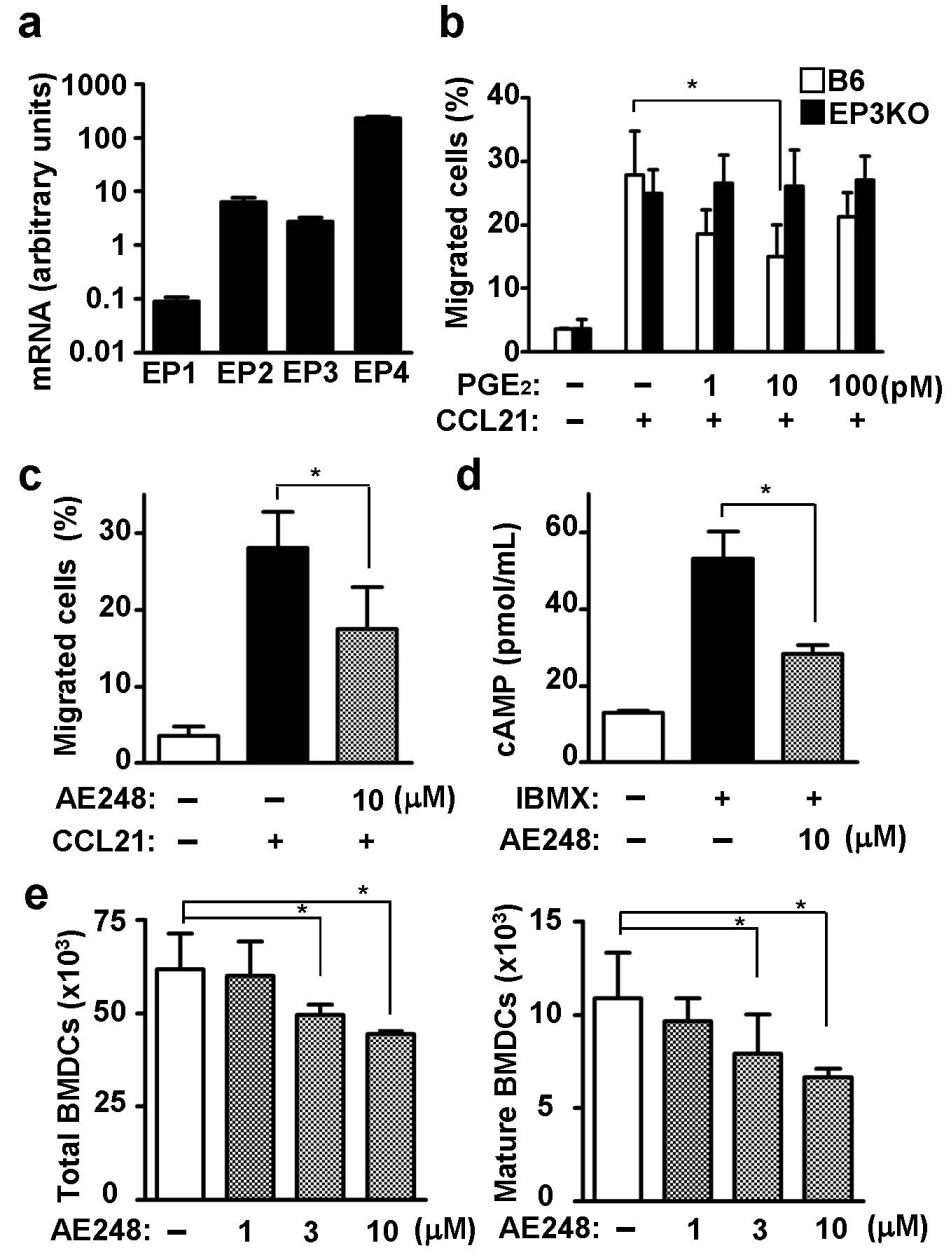
Inhibition of migration and maturation of BMDCs by EP3 agonist (AE248). (a) mRNA Expression of the four EP subtypes in BMDCs. (b) Low dose PGE_2_ attenuated the migration of BMDCs. BMDCs were treated with 0, 1, 10 and 100 pM PGE_2_ and applied to a transwell. 100 ng/mL CCL21 was administrated to the lower chamber. Migrated BMDCs were identified as MHC class II^+^ CD11c^+^ subset in the lower chamber. The % input was calculated as follows: (the number of BMDCs migrated into the lower chamber)/(the number of BMDCs applied into the upper chamber) *100. (c) EP3 agonist attenuated the CCL21-induced migration of BMDCs. BMDCs were treated with 10 μM AE248 for 2 days and applied to a transwell. The migration assay was carried out the same way as previously descried. (d) Intracellular cAMP concentration. BMDCs were treated with 0.5 mM IBMX for15min prior to EP3 agonist treatment (30min). (e) The number of total (CD86^high^ MHC class II ^middle to high^) and mature (CD86^high^ MHC class II ^high^) BMDCs were counted after EP3 agonist incubation for 2 days (n=6). Each data represents the mean + SD. *p<0.05.

We first confirmed the expression levels of EP subtypes in bone marrow-derived DCs (BMDCs) by reverse transcription-PCR. The expression level of EP3 mRNA was the second to third among the four EP subtypes. It was about one hundredths of the level of EP4, which was the most abundant in BMDCs (Figure 1a). However, we speculated that EP3 potentially modulates the functions of cutaneous DCs because the binding affinity of EP3 for PGE_2_ is much higher than that of EP4 [21].

### EP3 agonist inhibited migration and maturation of BMDCs *in vitro*

PGE_2_ is known to promote the migration of cutaneous DCs to draining lymph nodes in an EP4-dependent manner during cutaneous inflammatory conditions when the local concentration of PGE_2_ is high (~10 μM) [10]. Since the binding affinity of EP3 is much higher than that of EP4 [21], we hypothesized that PGE_2_-EP3 signaling may function when the local concentration of PGE_2_ is low (pM scale), for example, under the steady states or upon innocuous stimuli. Therefore, to validate the physiological significance, we examined the effect of low-dose PGE_2_ on the chemotactic activity of BMDCs to CCL21, one of the chemokines that induce the homing of DCs to draining lymph nodes. We found that as low as 10 pM of PGE_2_ reduced the chemotaxis of BMDCs to CCL21 when the concentration of CCL21 is 30 and 100 ng/mL, but not 300 ng/mL (Figure 1b and Figure S1). These findings suggest that PGE_2_ may have opposite effects on DC functions, possibly through EP3 signaling, which is consistent with the cAMP-lowering effect of EP3, differing from EP4. Intriguingly, when the dose of CCL21 was high, the inhibitory effect of PGE_2_ was not observed. Therefore, although the inhibitory effect of PGE_2_ on BMDC chemotaxis was about 50%, we assume that this inhibitory mechanism is important for fine-tuning of skin homeostasis.

Thus, we investigated the effect of EP3 signaling on DC migration responding to CCL21. We used BMDCs instead of cutaneous DCs to exclude the possible influences of the contaminating EP3-sensitive epidermal keratinocytes. We placed BMDCs in the upper chamber of Transwell^®^ and counted the number of major histocompatibility complex (MHC) class II^+^ CD11c^+^ BMDCs that migrated to the lower chamber filled with CCL21. The addition of the EP3 agonist to the upper chamber inhibited the migration of BMDCs (Figure 1c). We measured the amount of intracellular cAMP in the presence or absence of the EP3 agonist after the treatment of 0.5 μM 3-isobutyl-1-methylanthine (IBMX), which inhibits endogenous phosphodiesterase to fix the amount of the intracellular cAMP. We found that the EP3 agonist reduced the amount of cAMP to 50% of the IBMX treatment group (Figure 1d).

We also examined the effect of EP3 signaling on BMDC maturation. We cultured BMDCs with or without the EP3 agonist for 2 days and counted the MHC class II^high^ and CD86^high^ populations, which correspond to the mature BMDCs. The number of total and mature BMDCs was significantly reduced in the presence of the EP3 agonist (Figure 1e).

### EP3 agonist inhibited migration and maturation of LCs *in vitro*

**Figure 2:**
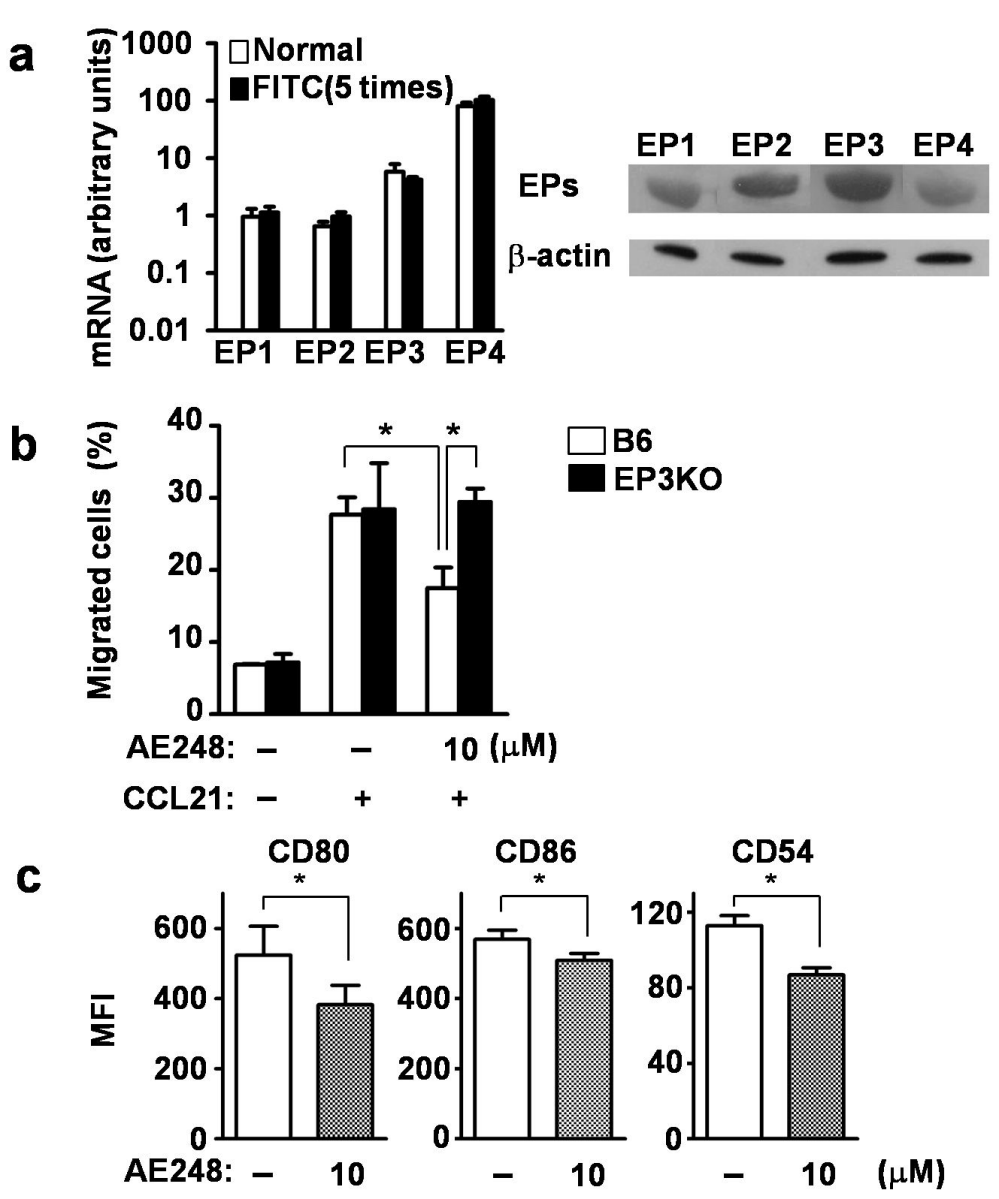
Inhibition of migration and maturation of LCs by EP3 agonist (AE248). (a) Expression of the four EP subtypes in CD11c^+^ epidermal cell suspensions (Langerhans cells) (Left; mRNA, Right; protein). (b) LCs treated with EP3 agonist for 24 hours were applied to the upper chamber of a transwell. After 3 hours incubation, the numbers of MHC class II ^+^ CD11c^+^ cells in the lower chamber including 100 ng/mL CCL21 were counted. White and black bars indicates the data from B6 and EP3KO mice, respectively (n=3). (c) Expression of maturation markers (CD80, CD86 and CD54) on LCs were analyzed by FACS 24 hours after EP3 agonist treatment (n=3). *p<0.05. MFI, mean fluorescence intensity.

To evaluate the effect of EP3 signaling on cutaneous DCs, we prepared epidermal LCs as a representative of cutaneous DCs that play an important role in the development of epicutaneous sensitization [22]. Consistent with the finding in BMDCs, EP3 mRNA was observed in LCs under the steady and repeated hapten applied conditions by reverse transcription-PCR (Figure 2a, left). EP3 protein was also detected in LCs in the steady state of C57BL/6 (B6) mice by western blot analysis (Figure 2a, right). We then prepared epidermal cell suspensions from ears of B6 and EP3KO mice, and incubated them for 24 hours with or without the EP3 agonist. These cells were applied to the upper chambers of Transwell^®^ with lower chambers containing CCL21. We then counted the number of migrated LCs as CD11c^+^ MHC class II^+^ cells in the lower chamber after 3 hours. The migration of B6-derived LCs was inhibited by the EP3 agonist, whereas that of EP3KO-derived LCs was not affected (Figure 2b), indicating that EP3 signaling specifically inhibits the migration of LCs. To examine the effect of the EP3 agonist on the maturation of LCs, we cultured epidermal cell suspensions from B6 mouse ears for 24 hours with or without the EP3 agonist. The expression levels of co-stimulatory molecules, such as CD80 and CD86, and adhesion molecule CD54 were decreased by the addition of the EP3 agonist (Figure 2c). Taken together, EP3 signaling suppressed the migration and maturation of LCs *in vitro*.

### EP3 restricted migration of cutaneous DCs under suboptimal antigen stimulation *in vitro*

**Figure 3:**
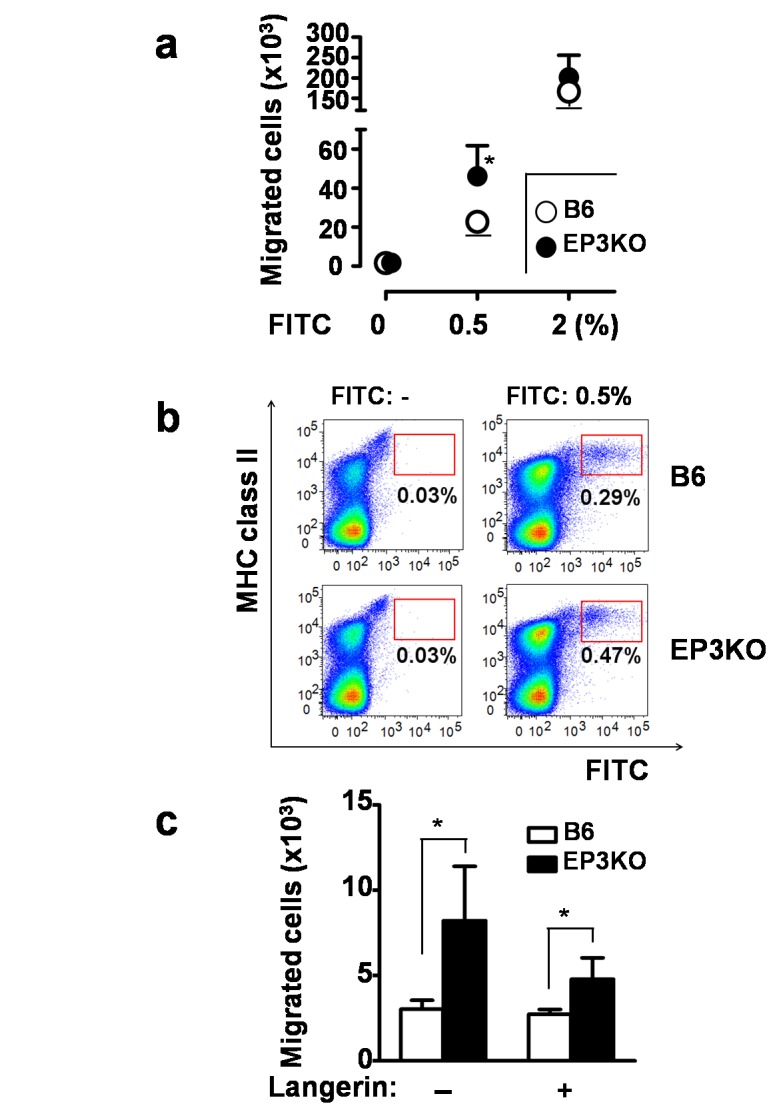
Enhanced cutaneous DC migration in EP3KO mice. (a) The number of FITC^+^ MHC class II^+^ DCs in the draining lymph nodes from EP3KO and WT mice 96 hours after application of 0.5 or 2% FITC (n=4). (b) Representative flow cytometry of lymph node cells. (c) The numbers of FITC^+^ LCs (FITC^+^ Langerin^+^ cells) or dermal DCs (FITC^+^ Langerin^-^ cells) in the draining lymph nodes of the mice after application of 0.5% FITC. White and black columns indicate B6 and EP3KO mice, respectively (n=4). Each data represents the mean + SD. *p<0.05.

To further test our hypothesis that PGE_2_-EP3 signaling functions *in vivo*, we applied different doses of fluorescein-5-isothiocyanate (FITC) onto the abdominal skin of mice under the steady state or upon innocuous/mild stimuli. Then we counted the number of FITC^+^ MHC class II^+^ cutaneous DCs that migrated from the skin to the draining lymph nodes after 96 hours of immunization (Figure 3a and b). At the dose of 2% that is commonly used in this FITC assay, there was no significant difference in the degree of migration between EP3KO and B6 mice in accordance with our previous report [10]. Intriguingly, however, the number of migrated FITC^+^ cells was apparently increased in EP3KO mice when the concentration of FITC was 0.5% (Figure 3a and b).

We measured the PGE_2_ levels in FITC-applied skin. We collected the skin samples using punch biopsy (8 mm in diameter) 24 hours after 0.5% FITC application. Skin sections were homogenized in PBS and PGE_2_ levels in the supernatant were measured with a PGE_2_ EIA kit (Cayman Chemical). PGE_2_ levels with or without FITC applications were comparable between B6 and EP3KO mice (Table S1). These data suggest that the difference of migrated DCs between B6 and EP3KO mice depends not on the expression level of PGE_2_ in the skin, but on the EP3 signaling after binding of PGE_2_ to EP3 receptor. This indicates that EP3 attenuates the migration of cutaneous DCs only when the antigen is applied at a suboptimal dose. We further examined the number of migrated FITC^+^ cutaneous DCs by dividing into Langerin^+^ DCs (including LCs and Langerin^+^ dermal DCs) and Langerin^-^ dermal DCs, and found that both subsets were increased in EP3KO mice (Figure 3c). Therefore, EP3 signaling restrains the migration of cutaneous DCs when the external stimuli are subtle.

### Suboptimally primed CHS response was inhibited by EP3

We examined whether the restriction on cutaneous DC functions by EP3 is also involved in the CHS response that is a murine allergic contact dermatitis model. CHS is a cascade of sequential events that starts with the sensitization phase and is terminated by the priming of hapten-specific T cells. Re-exposure of the hapten initiates the second phase of CHS, which is called the elicitation phase. It is well known that cutaneous DCs play a pivotal role in the sensitization phase by migrating into the draining lymph nodes to activate T cells.

**Figure 4:**
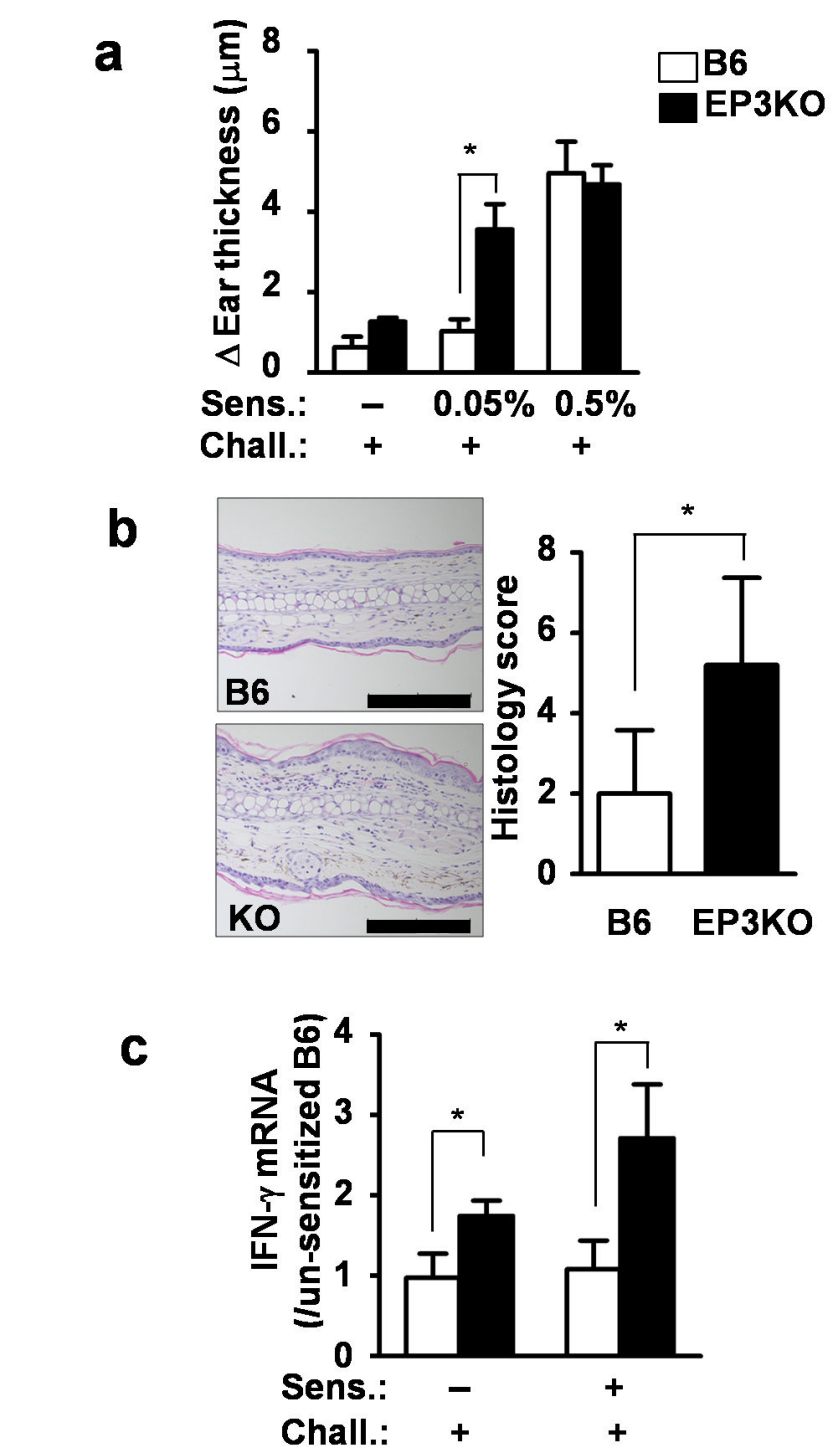
Enhancement of cutaneous immune response in EP3KO mice. (a) B6 and EP3KO mice (n=5 per group) were sensitized with 0 (un-sensitized), 0.05 or 0.5%-DNFB and the ear swelling was measured 24 hours after challenge with 0.3%-DNFB. (b) Hematoxylin-eosin staining of ears after the challenge. Scale bar indicates 200 µm. The total histology score was calculated as the sum of four elements (inflammation, neutrophils, edema and epithelial hyperplasia). (c) IFN-γ mRNA in the draining lymph nodes of the un-sensitized and 0.05%-DNFB-sensitized mice. Draining lymph nodes were collected after the measurement of ear swelling (n=5). Each data represents the mean + SD. *p<0.05. sens, sensitization; chall, challenge.

By sensitizing abdomens with 50 µl of 0.5% 2,4-dinitro-1-fluorobenzene (DNFB) five days before, we could elicit ear swelling in both B6 and EP3KO mice after 24 hours of challenge with 20 µl of 0.3% DNFB. Importantly, sensitization with 0.05% DNFB was suboptimal for normal B6 mice and did not elicit ear swelling after the challenge. However, the same sensitization did elicit ear swelling in EP3KO mice at a comparable level to that of optimally sensitized mice (Figure 4a). Histological analysis confirmed significant inflammation in the ears of 0.05% DNFB-sensitized EP3KO mice (Figure 4b and Table S2). Accordingly, there was approximately twice the amount of IFN-γ transcripts in the draining lymph nodes of 0.05% DNFB-sensitized and challenged EP3KO mice compared to that of B6 mice (Figure 4c). Moreover, careful observation revealed that EP3KO mice exhibited mildly enhanced ear swelling and a mild increase in the amount of IFN-γ transcripts in the draining lymph nodes after the challenge even without sensitization (Figure 4a and c). This observation strongly indicates that EP3 engagement constitutively operates against undesirable CHS to a suboptimal dose of antigen exposure.

In addition, to assess the contribution of PGE_2_-EP3 signaling on DCs/LCs circulation, we measured the quantifications of the frequencies of DCs/LCs in skin and lymph nodes using B6 and EP3KO mice in the steady state. The frequency of LC in epidermal cell suspension from the earlobes, and resident DCs (MHC class II^middle^ CD11c^high^) and migrated DCs (MHC class II^high^ CD11c^middle^) in lymph nodes were comparable between B6 and EP3KO mice in the steady state (Table S3). Therefore, PGE_2_-EP3 signaling may not contribute DCs/LCs circulation between skin and lymph node in the steady state.

**Figure 5:**
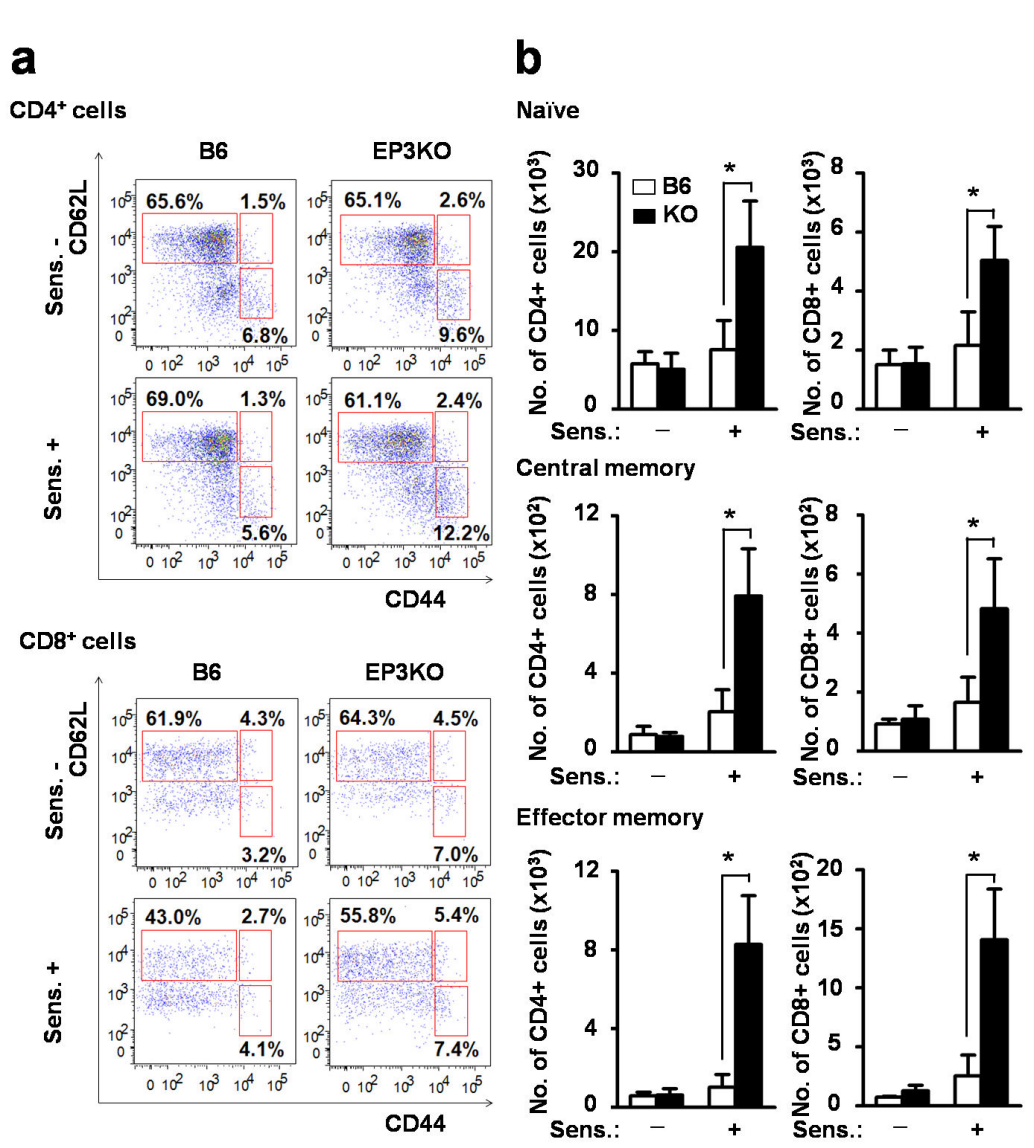
Increased the number of T cell subsets in draining lymph nodes in EP3KO mice. Draining lymph nodes were collected the mice of 5 days after the DNFB or vehicle application mice. (a) Representative flow cytometric plots. (b) The numbers of CD44^-^ CD62L^+^ naïve (upper panel), CD44^+^CD62L^+^ central memory (middle panel), and CD44^+^CD62L^-^ effector memory (lower panel) subsets of CD4^+^ T cells (left panels) and CD8^+^ T cells (right panels) of B6 and EP3KO mice were counted and indicated by white and black bars, respectively (n=6). Each data represents the mean + SD. *p<0.05.

To further characterize the effect of EP3 signaling during the sensitization phase of CHS, we analyzed T cell subsets in the lymph nodes. After the challenge, the number of CD44^-^ CD62L^+^ naïve T cells, CD44^+^CD62L^+^ central memory T cells, and CD44^+^CD62L^-^ effector memory T cells were markedly increased in the lymph nodes of EP3KO mice that were suboptimally sensitized 5 days before (Figure 5a and b). However, the proportion of each subset did not show significant difference between EP3KO mice and B6 mice (Figure 5a). Therefore, together with the previous report that EP3 is not expressed in T cells [20], we speculated that the effect of EP3 is mainly seen in the migration and maturation of cutaneous DCs but not in the development of T cell subsets.

## Discussion

Thus far, there have been many reports on the activators of cutaneous DCs to initiate CHS, such as tumor necrosis factor, IL-1, and IL-18 [3], however, it remains unclear how the homeostasis of cutaneous DCs is maintained in relation to CHS. Our results have demonstrated that PGE_2_-EP3 signaling plays a unique role in the regulation of the CHS response. PGE_2_-EP4 signaling promotes the migration and maturation of cutaneous DCs, thereby initiating the CHS response on exposure to antigens. On the contrary, the PGE_2_-EP3 signaling functions in the opposite direction, balancing the cutaneous immune homeostasis in a subtle way. When the amount of antigens does not reach the threshold for appropriate immune responses, the PGE_2_-EP3 signaling actively limits the migration and maturation of cutaneous DCs through Gi protein to avoid unwanted inflammation. Once the antigen dose crosses the threshold, the PGE_2_-EP4 signaling axis overcomes the restriction by PGE_2_-EP3 signaling in order to switch the mode of cutaneous DCs toward activation.

**Figure 6:**
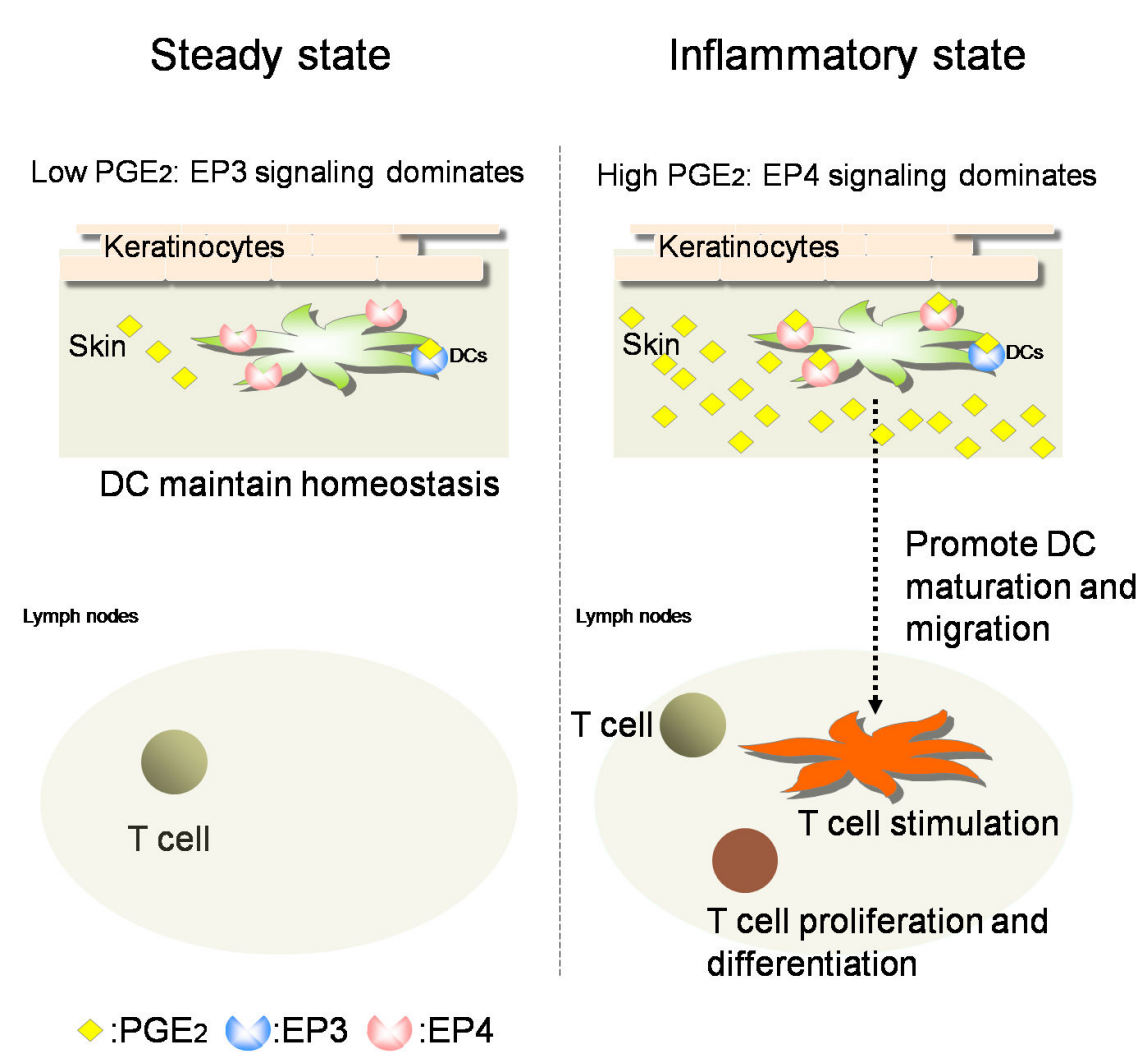
Hypothesis of the dual roles of PGE_2_ on cutaneous DCs. In the steady state when the concentration of PGE_2_ is low, endogenous PGE_2_ binds to EP3 preferentially (binding affinity of PGE_2_ to EP3 is higher than EP4), resulting in the prevention of impetuous immune responses to innocuous stimuli. On the other hand, in the inflammatory state, abundant PGE_2_ is produced by keratinocytes. High expression of PGE_2_ binds to EP4, which overcomes the inhibitory effect of EP3 signaling and promotes the maturation and migration of cutaneous DCs to initiate skin immune responses.

We do not yet know the molecular mechanism that determines which EP subtype will dominate in response to PGE_2_. As DCs express both EP3 and EP4, the cell surface expression level of EP3 and EP4 may be differentially regulated depending on the antigen dose. However, at the transcript level, the mRNA level of EP4 was at least tenfold higher than that of EP3 (see Figures 1a and 2b). Therefore, it is not easy to explain the mechanism simply by the expression level of these subtypes. Alternatively, there may be cross talk between EP3 and EP4. For example, EP3-coupling Gi may somehow over-rule EP4-coupling Gs under non-inflammatory conditions when the production of PGE_2_ is low. On the other hand, once DCs are exposed to a large dose of antigens, EP4-coupling Gs now dominates the relation (see Figure 6). In line with the above hypothesis, the binding affinity of EP3 for PGE_2_ is much higher than that of EP4 [21].

EP3 is the only prostanoid receptor that couples Gi and functions in a cAMP-inhibitory manner. Other prostanoid receptors work in an either Ca^2+^ stimulatory (EP1, FP, TP) or cAMP-stimulatory fashion (EP2, EP4, DP, IP). This multiplicity of EP subtypes makes PGE_2_ the most versatile prostaglandin *in vivo*. Here we revealed another unexpected dual role of PGE_2_ on the CHS response (see Figure 6). In the steady state, low-dose PGE_2_ limits migration and maturation of cutaneous DCs through EP3 to halt impetuous response to suboptimal stimuli. Thus, PGE_2_-EP3 axis seems to exhibit fine-tuning excessive skin inflammation by restricting DC functions. This limitation is easily cancelled under inflammatory state by high-dose PGE_2_, which now acts on EP4 to switch the state of cutaneous DCs to an activation mode.

The mechanism to initiate skin immune responses have been vigorously studied, but the mechanism how to keep skin homeostasis has not been revealed well. In this study, we focused on the role of DCs. On the hand, other possible candidates to maintain skin homeostasis include regulatory T cells (Tregs). In the absence of Tregs, mice lead to spontaneous skin inflammation [23] and enhanced CHS to hapten exposure [24–26]. It remains unclear whether PGE_2_-EP3 signaling on DCs modulates the induction of Tregs, which will be addressed in the future.

It has also been reported that PGE_2_-EP3 signaling suppressed conjunctivitis and airway inflammation by inhibition of epithelial cells and mast cells [27,28]. Since DCs are widely distributed to the tissues, PGE_2_-EP3 signaling may exhibit anti-inflammatory effects in other tissues, including the conjunctiva and respiratory tract. Therefore, to exploit this versatile PGE_2_-EP3/EP4 pathway can lead to a novel approach for treating skin inflammation, including allergic contact dermatitis.

## Materials and Methods

### Animals and reagents

BALB/c and B6 mice were purchased from Japan SLC (Hamamatsu, Japan). EP3KO mice were backcrossed more than ten times onto B6. Mice were maintained in specific pathogen–free facilities at the Kyoto University, and eight-to thirteen-week-old mice were used for experiments. All experimental procedures were approved by the institutional animal care and use committee of Kyoto University Graduate School of Medicine (Permit Number: 080150).

ONO-AE-248 (AE248), a selective EP3 agonist, was supplied by Ono Pharmaceutical Co, Ltd. (Osaka, Japan); the ligand-binding specificities of the compound for each PGE_2_ receptor subtype have been described [29]. AE248 was used at 10 μM for *in vitro* experiments. We purchased PGE_2_ from Cayman Chemical Company (Ann Arbor, MI, USA), DNFB from Nacalai Tesque (Kyoto, Japan), and FITC from Sigma-Aldrich (St. Louis, MO, USA). To remove endogenous prostanoids, indomethacin (Sigma-Aldrich) was applied at 10 μM for *in vitro* experiments.

### Cell preparation and cultures

RPMI 1640 (Sigma-Aldrich) containing 10% heat-inactivated fetal calf serum (Invitrogen, Carlsbad, CA, USA), 50 µM 2-mercaptoethanol, 2 mM L-glutamine, 1 mM nonessential amino acids, 1 mM sodium pyruvate, 100 units/ml penicillin, and 100 µg/ml streptomycin, was used as culture medium, complete RPMI (cRPMI).

Epidermal cell suspensions were obtained from the earlobes of mice with trypsin treatment [30]. LCs were purified from epidermal cell suspensions as CD11c^+^ cells (purity >70%) by means of auto MACS (Miltenyi Biotec, Auburn, CA, USA).

BMDCs were prepared as described [31]. In brief, 5 × 10^6^ bone marrow cells were cultured in 10 cm tissue culture dishes in 10 ml of cRPMI medium supplemented with 10 ng/ml recombinant murine GM-CSF (PeproTech, Rocky Hill, CT, USA) for 5 days. Loosely adherent cells were harvested at day 5 and incubated at 1 × 10^6^ cells/ml for another 2 days.

### Western Blotting

CD11c^+^ ECs (LCs) was homogenized in a RIPA buffer (Wako, Osaka, Japan) supplemented with a protease inhibitor mixture tablet (Roche, Basel, Switzerland). Protein supernatant was extracted after a 3000 rpm centrifugation at 4°C for 30 min. Ten μg of protein was then electrophoresed on a precast of NuPAGE Bis-4–12% Bis-Tris Mini gel (Invitrogen, Carlsbad, CA), and transferred onto a polyvinyl difluoride membrane. Membranes were blocked with 5% skim milk in PBS containing 0.1% Tween-20 for 1 hour at room temperature, and incubated with primary Abs against EP1 (1:500, Cayman Chemical), EP2 (1:200, Acris Antibodies, San Diego, CA, USA), EP3 (1:200, Cayman Chemical) and EP4 (1:200, Cayman Chemical) at 4°C overnight. Then, the membranes were incubated with horseradish peroxidase (HRP)-conjugated anti-rabbit antibodies (1:5000, Cell Signaling Technology, Boston, MA) for 1 hour at room temperature. The ECL Plus Western blot detection system (Amersham, Piscataway, NJ) were used for visualization. To reprobe β-actin, membranes were stripped using Restore^TM^ plus Western blot stripping buffer (Thermo scientific, Rockford, IL) for 15 minutes at room temperature. After washing, they were incubated with TBS with 5% skim milk (for 1 hour) and subsequently with the monoclonal β-actin antibody (Cell Signaling Technology).

### Flow cytometry

Cell suspensions were prepared from lymph nodes by mechanical disruption on 70 μm nylon cell strainers (BD Falcon, San Jose, CA, USA). For flow cytometry, cells were prepared and stained with antibodies (Abs) as described previously [32].

FITC, phycoerythrin (PE), PE-Cy5, PE-Cy7, allophycocyamin (APC), and biotin-conjugated anti-CD4, anti-CD8, anti-CD11c, anti-CD54, anti-CD62L, anti-CD80, anti-CD86, anti-Langerin (CD207), and anti-MHC class II mAbs were purchased from eBioscience (San Diego, CA, USA). For Langerin staining, cells were fixed and permeabilized with cytofix/cytoperm solution (BD Biosciences, San Jose, CA, USA), and stained with biotin-conjugated anti-Langerin Ab. Cells were collected with FACSCantoII or LSRFortessa (BD, Franklin Lakes, NJ, USA) and analyzed with FlowJo software (TreeStar, San Carlos, CA, USA).

### Quantitative reverse transcription-PCR

Total mRNA was extracted with the SVTotal RNA Isolation system (Promega, Madison, WI). Target gene expression was quantified in a two-step reverse transcription-PCR. cDNA was reverse transcribed from total RNA samples using the TaqMan RT reagents (Applied Biosystems, Foster City, CA, USA). Murine EP1, 2, 3, and 4 and IFN-γ (Assay ID: Mm00443097_m1, Mm00436051_m1, Mm00441045_m1, Mm00436053_m1, Mm01168134_m1) expressions were quantified using TaqMan Gene Expression Assay in the ABI PRISM 7000 sequence detection system (Applied Biosystems). As an endogenous reference for these PCR quantification studies, β-actin gene expression was measured using the TaqMan β-actin control reagents. The relative expression was calculated using the 2^-ΔΔCT^ method [33]. The expression of the target gene normalized to an endogenous reference and relative to a calibrator is given by the formula 2^-ΔΔCT^. Gene expression in untreated mice was used as a calibrator expression to calculate ΔΔCT.

### cAMP assay

We treated 1 × 10^6^ cells of BMDC with 0.5 μM IBMX (Wako, Osaka, Japan) for 15 minutes. These cells were further incubated with or without the EP3 agonist (10 μM) for 30 minutes, and cAMP levels in the culture supernatant were measured with ELISA (R&D Systems, MN, USA).

### Chemotaxis assay and FITC-induced cutaneous DC migration

BMDCs and epidermal cell suspensions were tested for transmigration across uncoated 5-µm Transwell^®^ filters (Corning Costar Corp., Corning, NY, USA) for 3 hours to 100 ng/mL CCL21 (R&D systems) in the lower chamber [32]. The number of MHC class II^+^ CD11c^+^ cells in the lower chamber was counted as migrating cells by flow cytometry. The percent input was calculated as follows: (the number of cells migrated into the lower chamber)/(the number of cells applied to the upper chamber) × 100.

For FITC-induced cutaneous DC migration, mice were painted on their shaved abdomen with 100 μL of 0.5% FITC or 200 μL of 2% FITC dissolved in a 1:1 (v/v) acetone/dibutyl phthalate (Sigma-Aldrich) mixture, and the number of migrated cutaneous DCs into draining inguinal and axillary lymph nodes was enumerated by flow cytometry.

### DNFB-induced CHS model

For the CHS model, mice were immunized by application of 50 μL of 0.5 or 0.05% (wt/v) DNFB in 4:1 (v/v) acetone/olive oil to their shaved abdomens on day 0. They were challenged on the right ear on day 5 with 20 μL of 0.3% (wt/v) DNFB^21^. Ear thickness was measured before and 24 hours after the challenge to assess inflammation.

To examine mRNA expression and histological examination, ears were collected after ear thickness measurement. For histological examination, tissues were fixed with 10% formalin in phosphate buffered saline and then embedded in paraffin. Sections with a thickness of 4 μm were prepared and subjected to staining with hematoxylin-eosin. The histological findings were evaluated for the severity and character of the inflammatory response using a subjective grading scale as reported previously [34].

### Statistical analysis

Data were analyzed using an unpaired Student’s *t*-test or Dunnett’s multiple comparison tests. A *p* value of less than 0.05 was considered to be significant. Unless otherwise indicated, data are presented as means + standard deviations (SDs).

## Supporting Information

Figure S1 Effects of PGE_2_ on migration of BMDCs. Effects of PGE_2_ on migration of BMDCs to CCL21 BMDCs were treated with 0, 1, 10 and 100 pM PGE_2_ and applied to a transwell. 30 ng/mL CCL21 (left panel) and 300 ng/mL CCL21 (right panel) were administrated to the lower chamber. Migrated BMDCs were identified as MHC class II^+^ CD11c^+^ subset in the lower chamber. The % input was calculated as follows: (the number of BMDCs migrated into the lower chamber)/(the number of BMDCs applied into the upper chamber) *100 (n=4-5). Each data represents the mean + SD. *p<0.05.
(TIF)

Table S1 PGE_2_ concentration in the skin. B6 and EP3KO mice were painted with 0.5% of FITC. The skin samples were collected 24 hours after 0.5% FITC application using skin punch biopsies (8 mm in diameter). The skin samples were homogenized in 1 mL of PBS, and PGE_2_ levels in the supernatant were measured with a PGE_2_ EIA kit (Cayman Chemical). Data indicate the mean ± SD of 3 mice.
(DOC)

Table S2 Histological evaluation of **CHS**.Mice were sensitized with 0.05% of DNFB and challenged. Histology scores are calculated as the sum of four elements (inflammation, neutrophils, edema, and epithelial hyperplasia). Data indicate the mean ± SD of 5 mice.
(DOC)

Table S3 The frequency of DCs and LCs in the skin draining lymph nodes and skin. Earlobes and inguinal lymph nodes were collected from B6 and EP3KO mice. Epidermal cell suspensions and lymph node cell suspensions were prepared and subjected to FACS analysis for quantifications of the frequencies of resident and migratory DCs and LCs. Data indicate the mean ± SD of 3 mice.
(DOC)
