# Supplementary figures and images for: Prostaglandin E2-EP3 Axis in Fine-Tuning Excessive Skin Inflammation by Restricting Dendritic Cell Functions
Source: PLoS One. 2013 Jul 29;8(7):e69599. doi: 10.1371/journal.pone.0069599 (PMC3726673; doi:10.1371/journal.pone.0069599)

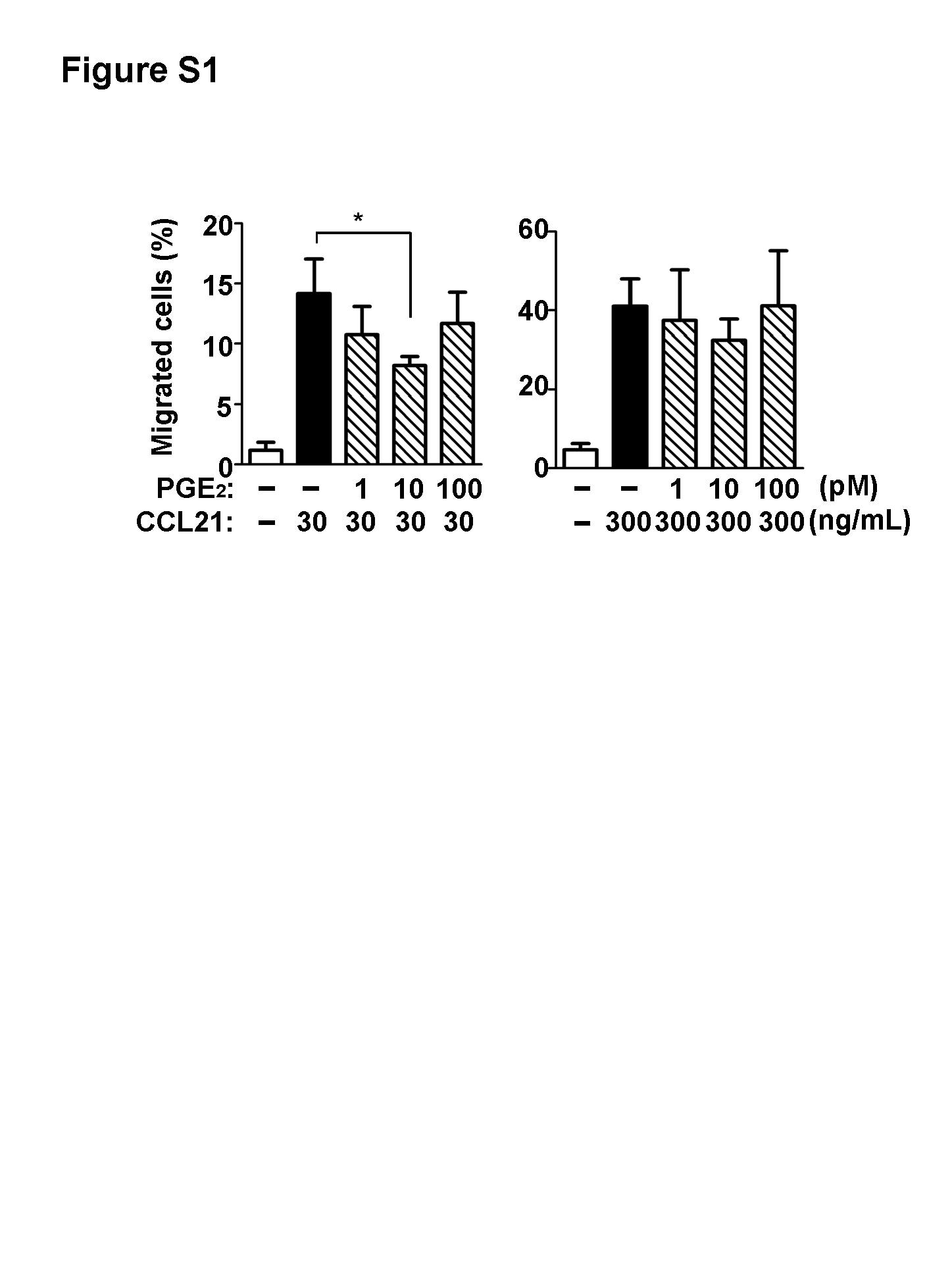

Supplement: Figure S1 — Effects of PGE2 on migration of BMDCs to CCL21 BMDCs were treated with 0, 1, 10 and 100 pM PGE2 and applied to a transwell. 30 ng/mL CCL21 (left panel) and 300 ng/mL CCL21 (right panel) were administrated to the lower chamber. Migrated BMDCs were identified as MHC class II+ CD11c+ subset in the lower chamber. The % input was calculated as follows: (the number of BMDCs migrated into the lower chamber)/(the number of BMDCs applied into the upper chamber) *100 (n=4-5). Each data represents the mean + SD. *p<0.05. (TIF) [file pone.0069599.s001.tif]
