# Supplementary material for: Prostaglandin E2-EP3 Axis in Fine-Tuning Excessive Skin Inflammation by Restricting Dendritic Cell Functions
Source: PLoS One. 2013 Jul 29;8(7):e69599. doi: 10.1371/journal.pone.0069599 (PMC3726673; doi:10.1371/journal.pone.0069599)
Supplement: Table S1 — B6 and EP3KO mice were painted with 0.5% of FITC. The skin samples were collected 24 hours after 0.5% FITC application using skin punch biopsies (8 mm in diameter). The skin samples were homogenized in 1 mL of PBS, and PGE2 levels in the supernatant were measured with a PGE2 EIA kit (Cayman Chemical). Data indicate the mean ± SD of 3 mice. (DOC) [file pone.0069599.s002.doc]

**Table S1. PGE_2_ concentration in the skin**

B6 and EP3KO mice were painted with 0.5% of FITC. The skin samples were collected 24 hours after 0.5% FITC application using skin punch biopsies (8 mm in diameter). The skin samples were homogenized in 1 mL of PBS, and PGE_2_ levels in the supernatant were measured with a PGE_2_ EIA kit (Cayman Chemical). Data indicate the mean ± SD of 3 mice.

|  | B6 mice | EP3KO mice |
| --- | --- | --- |
| Control skin | 398 ± 49 pg/mL | 398 ± 86 pg/mL |
| 0.5% FITC painted skin | 2110 ± 317 pg/mL | 1962 ± 777 pg/mL |
