# Supplementary material for: Prostaglandin E2-EP3 Axis in Fine-Tuning Excessive Skin Inflammation by Restricting Dendritic Cell Functions
Source: PLoS One. 2013 Jul 29;8(7):e69599. doi: 10.1371/journal.pone.0069599 (PMC3726673; doi:10.1371/journal.pone.0069599)
Supplement: Table S2 — CHS.Mice were sensitized with 0.05% of DNFB and challenged. Histology scores are calculated as the sum of four elements (inflammation, neutrophils, edema, and epithelial hyperplasia). Data indicate the mean ± SD of 5 mice. (DOC) [file pone.0069599.s003.doc]

**Table S2. Histological evaluation of CHS**

Mice were sensitized with 0.05% of DNFB and challenged. Histology scores are calculated as the sum of four elements (inflammation, neutrophils, edema, and epithelial hyperplasia). Data indicate the mean ± SD of 5 mice.

|  | B6 mice | EP3KO mice |
| --- | --- | --- |
| Inflammation | 0.20 ± 0.45 | 0.80 ± 0.45 |
| Neutrophils | 0.40 ± 0.55 | 0.80 ± 0.45 |
| Edema | 0.80 ± 0.45 | 1.60 ± 0.55 |
| Epithelial hyperplasia | 0.60 ± 0.55 | 2.00 ± 1.00 |
| Histology score (total) | 2.00 ± 1.58 | 5.20 ± 2.17 |
