# Supplementary material for: Prostaglandin E2-EP3 Axis in Fine-Tuning Excessive Skin Inflammation by Restricting Dendritic Cell Functions
Source: PLoS One. 2013 Jul 29;8(7):e69599. doi: 10.1371/journal.pone.0069599 (PMC3726673; doi:10.1371/journal.pone.0069599)
Supplement: Table S3 — Earlobes and inguinal lymph nodes were collected from B6 and EP3KO mice. Epidermal cell suspensions and lymph node cell suspensions were prepared and subjected to FACS analysis for quantifications of the frequencies of resident and migratory DCs and LCs. Data indicate the mean ± SD of 3 mice. (DOC) [file pone.0069599.s004.doc]

**Table S3. The frequency of DCs and LCs in the skin draining lymph nodes and skin**

Earlobes and inguinal lymph nodes were collected from B6 and EP3KO mice. Epidermal cell suspensions and lymph node cell suspensions were prepared and subjected to FACS analysis for quantifications of the frequencies of resident and migratory DCs and LCs. Data indicate the mean ± SD of 3 mice.

| Locations | DCs/LCs | Gate | B6 mice | EP3KO mice |
| --- | --- | --- | --- | --- |
| Lymph nodes | Resident DCs | MHC class II^middle^ CD11c^high^ | 0.86 ± 0.18 % | 0.79 ± 0.13 % |
| Lymph nodes | Migratory DCs | MHC class II^high^ CD11c^middle^ | 0.82 ± 0.26 % | 0.60 ± 0.10 % |
| Epidermal cell suspensions | LCs | MHC class II^+^ CD11c^+^ | 3.0 ± 1.0 % | 2.7 ± 0.7 % |
